# Fast-responding functional DNA superstructures for stimuli-triggered protein release†

**DOI:** 10.1039/d1sc00795e

**Published:** 2021-05-07

**Authors:** Yuxin Zhang, Qiang Zhang, Fang Cheng, Yangyang Chang, Meng Liu, Yingfu Li

**Affiliations:** School of Environmental Science and Technology, Key Laboratory of Industrial Ecology and Environmental Engineering (Ministry of Education), Dalian University of Technology Dalian 116024 China mliu@dlut.edu.cn; School of Bioengineering, Dalian University of Technology Dalian 116024 China; School of Chemical Engineering, Dalian University of Technology Dalian 116024 China; Department of Biochemistry and Biomedical Sciences, McMaster University 1280 Main Street West Hamilton Ontario L8S4K1 Canada

## Abstract

Strategies that speed up the on-command release of proteins (*e.g.*, enzymes) from stimuli-responsive materials are intrinsically necessary for biosensing applications, such as point-of-care testing, as they will achieve fast readouts with catalytic signal-amplification. However, current systems are challenging to work with because they usually exhibit response times on the order of hours up to days. Herein, we report on the first effort to construct a fast-responding gating system using protein-encapsulating functional DNA superstructures (denoted as protein@3D DNA). Proteins were directly embedded into 3D DNA during the one-pot rolling circle amplification process. We found that the specific DNA–DNA interaction and aptamer–ligand interaction could act as general protocols to release the loaded proteins from 3D DNA. The resulting gating system exhibits fast release kinetics on the order of minutes. Taking advantage of this finding, we designed a simple paper device by employing protein@3D DNA for colorimetric detection of toxin B (*Clostridium difficile* marker). This device is capable of detecting 0.1 nM toxin B within 16 minutes.

## Abstract 2

A stimuli-responsive gating system enabled by protein@3D DNA was engineered, which allows controlled protein release in a fast-responsive manner.

## Graphical abstract

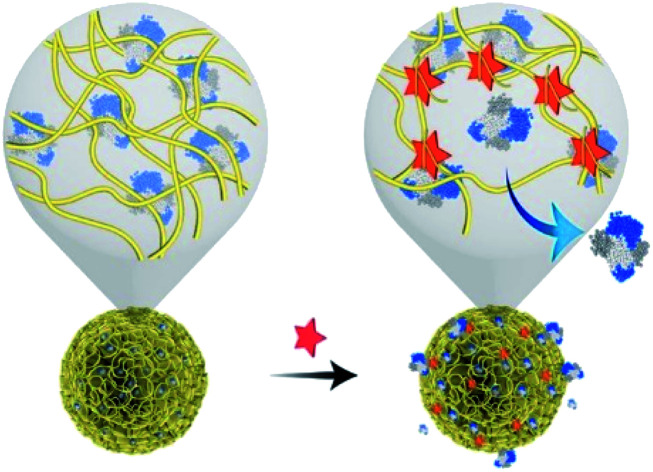

## Introduction

Learning from Mother Nature has inspired the design of smart, intelligent or stimuli-responsive materials.^1^ Of great interest are engineered gated materials in which the release of entrapped chemical or biochemical species can be finely tuned by applying external stimuli.^2^ This on-demand release feature designates gated materials as ideal platforms in biosensing, diagnostic, bioimaging and targeted drug delivery.^3^ A wide range of cargos from small molecules (*e.g.*, dyes, fluorophores, and redox-active agents) to macromolecules (*e.g.*, proteins, oligonucleotides, and nucleic acids) can be loaded into mesoporous silica (MPS) materials, metal–organic framework nanoparticles (NMOFs) and micro/nanocapsules.^1–3^ Upon exposure to different types of physical (light, temperature, ultrasound, and magnetism), chemical (pH, cations, anions, and small organic molecules) and biochemical (enzymes, and biomolecules) stimuli, it generally takes minutes, hours, even days, for the release of loaded cargos to reach equilibrium, depending on the strength and nature of the interactions between cargos and the support.^4^ For biosensing applications, it would be intrinsically necessary to speed up the release process, especially for bioactive protein enzymes, as they will allow the catalysis-driven signal-amplification for point-of-care testing.^5^ However, current gated systems usually operate over a period of hours and days during the controlled release of protein enzymes. For example, Lin *et al.*^4*c*^ reported a MPS nanoparticle-based system for the release of cytochrome *c*: a sigmoidal release curve was observed over a period of 25 h. Chu *et al.*^4*d*^ developed biomineralized ZIF-8 nanoparticles encapsulating proteins as a novel platform for controlled release of proteins, where acidic environments induced complete release of proteins in 2 h. To the best of our knowledge, no gating systems have been reported for the controlled protein release in a fast-responsive manner, or with response times on the order of minutes.

DNA nanostructures provide promising scaffolds to organize biomolecules with high programmability.^6^ Recently, self-assembled functional DNA superstructure (denoted as 3D DNA) has been used for *in situ* encapsulation of proteins through multiple weak interactions.^7^ Based on its highly ordered and flexible porous structure,^8^ we hypothesize that 3D DNA may present a novel class of gated materials for controlled cargo release in biosensing, particularly if it can be engineered to incorporate versatile functional DNAs (*e.g.*, DNA aptamers).

Herein, we report a fast-responding gating system enabled by protein-loaded 3D DNA (denoted as protein@3D DNA), which allows the controlled release of loaded proteins within the minute time scale. We observe that the formation of a specific DNA duplex or an aptamer–ligand complex serves as a versatile means to trigger the fast release process, through breaking the initial weak interactions between the protein and the 3D DNA.

## Results and discussion

### Design and characterization of protein@3D DNA

**Fig. 1:**
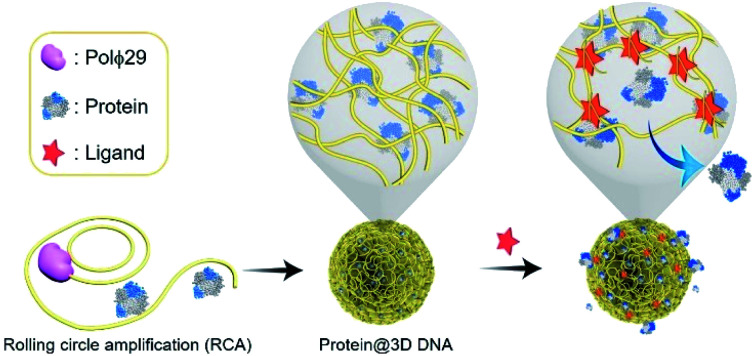
Schematic representation of the protein@3D DNA based gating system for on-command controlled release of loaded proteins. Synthesis of protein@3D DNA *via* one-pot rolling circle amplification (RCA). The controlled-release mechanism is based on the formation of duplex DNA units or aptamer–ligand complexes. Polϕ29 = ϕ29 DNA polymerase.

Controlled release of proteins from protein@3D DNA is shown in Fig. 1. Protein@3D DNA is synthesized in two steps (see experimental details in the ESI†): (1) the use of ϕ29 DNA polymerase (Polϕ29), a circular template (Ct) and a DNA primer (Dp) to encapsulate proteins into 3D DNA *via* one-pot rolling circle amplification (RCA, Fig. S1†);^9^ (2) isolation of protein@3D DNA by a centrifugal separation method.

**Fig. 2:**
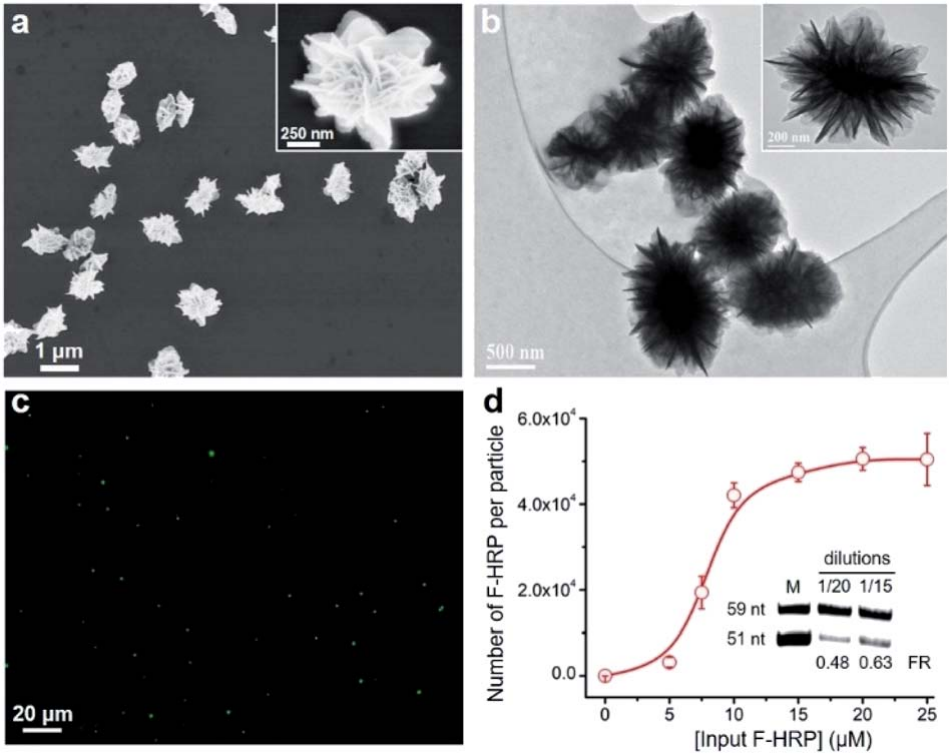
(a) SEM and (b) TEM images of HRP@3D DNA. (c) CFM image of F-HRP@3D DNA. λex = 488 nm. (d) Input F-HRP concentrations *vs.* number of F-HRP molecules per particle. Inset: denaturing polyacrylamide gel electrophoresis (dPAGE) analysis of the digested HRP@3D DNA obtained at varying dilutions. Top band: DNA internal control (59 nt). Bottom band: MA (51 nt). FR: ratio of fluorescence intensity of the 51 nt and 59 nt DNA bands.

We chose horseradish peroxidase (HRP) as a model protein, which has a molecular weight (MW) of 40 kDa and an isoelectric point (pI) of 7.2. Scanning electron microscopy (SEM) images in Fig. 2a revealed that HRP@3D DNA (0.73 ± 0.12 μm in diameter) exhibits the same flower-like hierarchical structures compared to pure 3D DNA (with a diameter of 1.02 ± 0.16 μm, Fig. S2†). Transmission electron microscopy (TEM) images indicated the formation of internal porous structures in HRP@3D DNA (Fig. 2b). To confirm that HRP molecules were indeed loaded into 3D DNA, fluorescently labeled HRP (F-HRP) was used for preparing F-HRP@3D DNA. Confocal fluorescence microscopy (CFM) images showed that monodisperse particles were homogenously luminescent following excitation (Fig. 2c). The loading capacity, *i.e.*, number of F-HRP per particle, gradually increased with increasing the input F-HRP concentration (Fig. 2d). The maximum loading capacity was determined to be (4.3 ± 0.3) × 10^4^ F-HRP molecules per particle (see the ESI† for details). The encapsulation efficiency of HRP was calculated to be ∼4%, independent of the input HRP concentration (Fig. S3†). In a control experiment, the simple physical mixtures of 3D DNA with HRP also resulted in the encapsulation of HRP into 3D DNA, producing a significantly decreased loading capacity of (0.3 ± 0.1) × 10^4^ F-HRP molecules per particle. Therefore, the multiple weak interactions such as the unique porous structure of 3D DNA, the abundance of Mg ions and phosphate backbones of DNA may facilitate the protein embedding during RCA. As 3D DNA from RCA contains many repeating sequence motifs,^9^ we converted it into monomeric amplicons (MA, 51 nucleotides) to quantify the total number of MA molecules per particle (Fig. 2d inset), yielding a value of (5.2 ± 1.1) × 10^6^.

We then examined the catalytic activity of HRP@3D DNA by a common method using hydrogen peroxide (H_2_O_2_) and 3,3′,5,5′-tetramethylbenzidine (TMB) as substrates (Fig. S4†). The loaded HRP in 3D DNA exhibited a decrease in the apparent *K*_m_ (Michaelis–Menten constant) compared to native HRP (Table 1, *p* < 0.001 and *p* < 0.05 comparing HRP with TMB and H_2_O_2_, respectively; Student's *t*-test), suggesting that DNA scaffolds improved the affinity of substrates to HRP. However, the obtained *k*_cat_ (turnover number) was lower for loaded HRP, implying that the encapsulation may lead to a decrease in its conformational flexibility for enzymatic catalysis.

**Table 1:** Kinetic data showing the Michaelis–Menten constant (*K*_m_) and turnover number (*k*_cat_) of native HRP and HRP@3D DNA

	Substrate	*K*_m_ a (mM)	*k*_cat_ a (s^−1^)
HRP	TMB	0.55 ± 0.06	134.74 ± 9.15
	H_2_O_2_	1.29 ± 0.12	720 ± 36.25
HRP@3D DNA	TMB	0.39 ± 0.06	44.74 ± 4.49
	H_2_O_2_	1.22 ± 0.14	77.88 ± 5.33

a Values are mean values ± SD from triplicate samples.

### DNA–DNA interaction-mediated protein release from protein@3D DNA

**Fig. 3:**
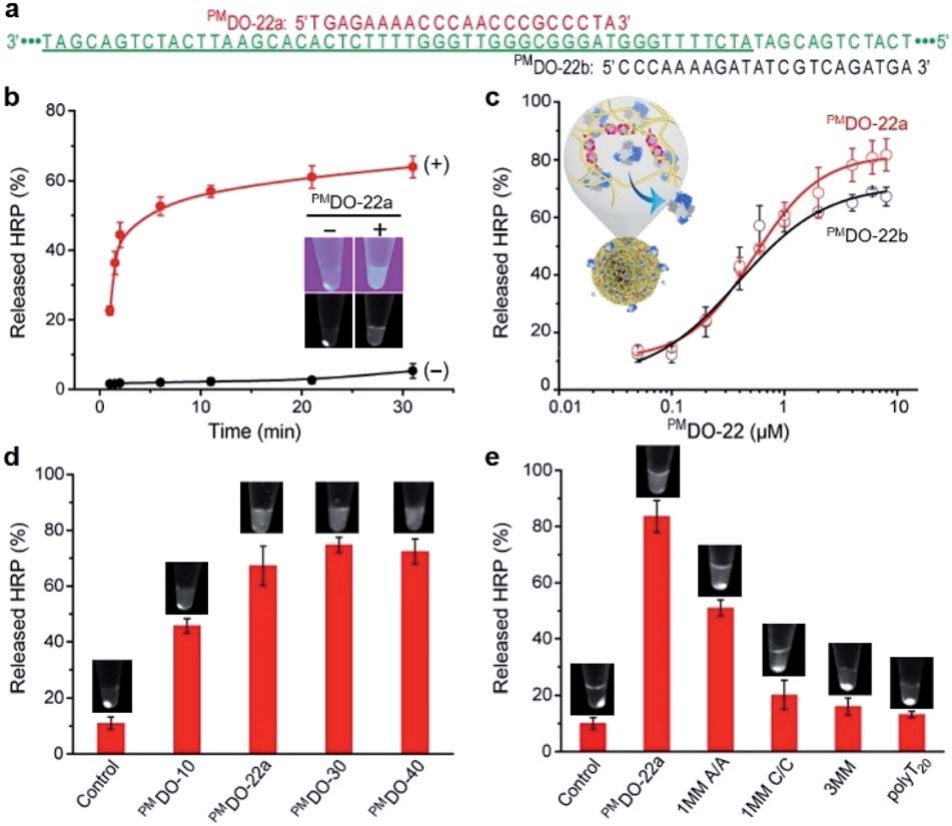
(a) The sequences of 3D DNA (green), ^PM^DO-22a (red) and ^PM^DO-22b (black). The MA sequence is shown in underlined. (b) Kinetic release of HRP from 3D DNA (∼10^7^ particles) in the absence (−) and presence (+) of 1 μM ^PM^DO-22a. Inset: photographs of the collected F-HRP@3D DNA without (−) and with (+) 1 μM ^PM^DO-22a under UV lights. The pictures were captured by a smartphone camera (top) and gel imaging system (bottom). (c) Relative released HRP (%) as a function of the concentration of ^PM^DO-22a and ^PM^DO-22b (*t* = 20 min). (d) The ^PM^DO length-dependent releases. [^PM^DO] = 2 μM. (e) Relative released HRP (%) in the presence of 6 μM certain DOs (*t* = 20 min). Photographs of F-HRP@3D DNA samples in the presence of indicated DOs are also shown. The pictures were captured by a gel imaging system. For the control samples, no ^PM^DO was added. All reactions were performed at room temperature in 50 μL of 1 × PBS buffer containing 5 mM MgCl_2_ (pH 7.4).

We next tested the ability of HRP@3D DNA (or F-HRP@3D DNA) to release HRP (F-HRP) in response to sequence-specific hybridization. A perfectly-matched DNA oligonucleotide containing 22 nucleotides (^PM^DO-22a) was designed to be complementary to part of MA within 3D DNA (Fig. 3a, red). Each sample was easily centrifuged to precipitate the F-HRP@3D DNA particles, followed by UV irradiation to determine the distribution of F-HRP in the supernatant and the precipitant. Without premixing with ^PM^DO-22a, F-HRP was found exclusively in the precipitant fraction, as evidenced by the strong luminescence signals at the bottom of tube. However, premixing 1 μM ^PM^DO-22a with the F-HRP@3D DNA results in the distribution of F-HRP in the supernatant fraction (Fig. 3b inset). Time-dependent release was recorded in Fig. 3b. The particles exhibited a negligible release (5.3 ± 2.1)% in 1 × PBS buffer (containing 5 mM MgCl_2_, pH 7.4) over a period of 30 min. In fact, this gated material showed that < 10% of HRP leaked after 20 days when stored at room temperature (Fig. S5†). However, the addition of 1 μM ^PM^DO-22a triggered the fast release of HRP, resulting in an observed rate constant (*k*_obs_) of (0.53 ± 0.04) min^−1^ and a final release of (63.9 ± 3.1)% at *t* = 30 min. In a control experiment, 3D DNA-adsorbed HRP particles exhibited a *k*_obs_ (0.35 ± 0.01) min^−1^ and a maximum release of (28.6 ± 2.8)% under same conditions (Fig. S6†). A proportional behavior between ^PM^DO-22a concentration (μM) and released HRP (%) was observed in Fig. 3c (red), and a maximum release of (80.8 ± 5.7)% was obtained by 6 μM ^PM^DO-22a. More specifically, by measuring the total amount of ^PM^DO-22a undergoing hybridization (Fig. S7†), we calculated that one HRP molecule could be released by about 15 bound ^PM^DO-22a. SEM result indicated the structural integrity of HRP@3D DNA after DNA hybridization (Fig. S8†).

We also investigated whether the location of DO binding site can influence the outcome of release. ^PM^DO-22b (Fig. 3a, black) was replaced by ^PM^DO-22a for this experiment. ^PM^DO-22b concentration-dependent release was also observed in Fig. 3c (black). We next examined if this controlled release required a specific size of DO. Three different sized DOs including ^PM^DO-10, ^PM^DO-30, and ^PM^DO-40, were tested. We observed a gradual increase in released HRP (%) with increasing size of DO (Fig. 3d). On the basis of these observations, we hypothesize that: when the target is absent, the protein was embedded into 3D DNA by multiple weak interactions; when the target is introduced, the 3D DNA prefers to form the stable 3D DNA-target duplex rather than the weak 3D DNA–protein complex, triggering the dissociation of the entrapped proteins.

To provide further evidence to support this scenario, we carried out two control experiments. First, various DOs including single base-pair mismatch with A/A or C/C mismatch (1MM A/A or 1MM C/C), three base-pair mismatch (3MM) and polyT20 were tested (sequences of all DNA species are provided in Table S1†). Fig. 3e demonstrated that the released HRP (%) was strongly dependent on the matching sequence for forming stable DNA duplex with 3D DNA. In the second experiment, HRP was covalently tethered to a 5′-amino-modified DNA (H_2_N-DNA) that was complementary to part of MA, denoted H_2_N-DNA-HRP (Fig. S9†). The resulting H_2_N-DNA-HRP@3D DNA (0.58 ± 0.11 μm in diameter, Fig. S10†), with a maximum loading capacity of (1.3 ± 0.3) × 10^5^ H_2_N-DNA-HRP per particle, exhibited a negligible release (<6%) even in the presence of 6 μM ^PM^DO-22a (Fig. S11†).

We investigated whether the hybridization-triggered HRP release was a general property of protein@3D DNA. Three fluorescently labeled proteins including recombinant streptococcus protein G (rSPG, 28 kDa), immunoglobulin G (IgG, 156 kDa) and cytochrome c (Cyt c, 12 kDa) were chosen (Table 2). Using the same method, we prepared rSPG@3D DNA, IgG@3D DNA and Cyt c@3D DNA (Fig. S12†). Note that the productivity of protein@3D DNA particles remained unaffected when different protein species were loaded, yielding a total number of ∼10^6^ particles per μL. As HRP (pI 7.2), rSPG (pI 4.8) and IgG (pI 5.5) carry neutral or net negative charge at pH 7.4, they exhibited similar loading capacities in the range of 10^4^ per particle. The isoelectric point of Cyt c is 10, making it positively charged at pH 7.4. 3D DNA is a negatively charged biopolymer due to its phosphate backbone. As a result, more Cyt c molecules were loaded into 3D DNA by this electrostatic potential difference. Upon addition of 1 μM ^PM^DO-22a, the total released rSPG, IgG and Cyt c molecules were determined to be (80.3 ± 0.2)%, (23.7 ± 0.4)%, and (5.3 ± 0.1)% at *t* = 30 min, respectively. These results allow us to conclude that: (1) the electrostatic attraction between protein and 3D DNA induces more loadings, but lower release is obtained; (2) the released protein increases as the size of protein decreases. Furthermore, IgG@3D DNA and rSPG@3D DNA exhibited *k*_obs_ values of (0.28 ± 0.16) min^−1^ and (0.59 ± 0.31) min^−1^, respectively (Fig. S13†). This result highlights the enticing advantage of protein@3D DNA in terms of fast release kinetics.

**Table 2:** Calculated total number of proteins per particle, maximum protein release, and *k*_obs_ for selected protein@3D DNA

Protein^a^	MW (kDa)	pI	Maximum loading capacity^b^ (×10^4^)	Protein release^b^ (%)	*k*_obs_ (min^−1^)
HRP	40	7.2	4.3 ± 0.3	63.9 ± 3.1	0.53 ± 0.04
rSPG	28	4.8	1.6 ± 0.7	80.3 ± 0.2	0.59 ± 0.31
IgG	156	5.5	1.9 ± 0.2	23.7 ± 0.4	0.28 ± 0.16
Cyt c	12	10	17 ± 2	5.3 ± 0.1	N.D.^c^

a FITC-labeled proteins.

b Data represent mean ± SD for three independent experiments. [^PM^DO-22a] = 1 μM; *t* = 30 min.

c N.D. = not detected.

### Aptamer–ligand interaction-mediated protein release from protein@3D DNA

**Fig. 4:**
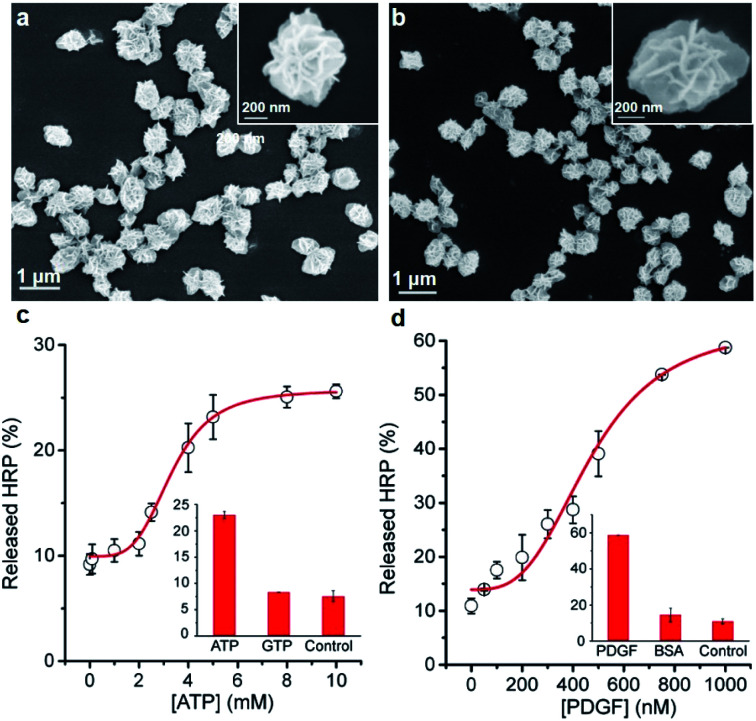
SEM images of HRP@3D DNA incorporated with concatemeric (a) ATP aptamers (0.58 ± 0.14 μm in diameter) and (b) PDGF aptamers (0.68 ± 0.13 μm in diameter). Relative released HRP (%) as a function of the concentration of (c) ATP and (d) PDGF at *t* = 10 min. Inset: relative released HRP (%) in the presence of non-intended molecules, GTP (5 mM) and BSA (1 μM).

Next, we examined whether the protein@3D DNA is compatible with functional DNA elements such as DNA aptamer, a synthetic oligonucleotide that can form well-defined tertiary structure with its cognate target.^10^ To test this hypothesis, we prepared two different F-HRP@3D DNA particles that contain well-characterized concatemeric aptamers known to bind adenosine triphosphate (ATP) and platelet-derived growth factor (PDGF),^11^ respectively (Fig. 4a and b). The total number of monomeric ATP aptamers and PDGF aptamers per particle were determined to be (2.9 ± 0.3) × 10^6^ and (3.9 ± 0.4) × 10^7^, respectively (Fig. S14†). It was observed that each particle provided a target concentration-dependent release of HRP (Fig. 4c and d). The particles treated with 5 mM ATP showed a HRP release of (25.7 ± 3.0)% at *t* = 10 min, whereas the PDGF-treated particles were able to produce (30.0 ± 0.5)% of HRP release by 0.4 μM PDGF. This result suggests that the release process is highly dependent on the target-binding affinity of the aptamer: the anti-ATP aptamer with a *K*_d_ (dissociation constant) of 10 μM (ref. 11*a*) and the anti-PDGF aptamer with a *K*_d_ of 0.1 nM.^11*b*^ As a result, higher ATP concentrations are required to induce the HRP release. Furthermore, both particles exhibited the fast *k*_obs_ values against ATP (0.18 min^−1^) and PDGF (0.08 min^−1^), respectively (Fig. S15†). Control experiments with a mutant HRP@3D DNA (in which aptameric sequences were mutated into random sequences) and other non-intended molecules (*i.e.*, GTP, BSA) demonstrated that the release was dependent on the specific aptamer sequence as well as the matching target for the aptamer (Fig. S16†). These results suggest that the formation of stable aptamer–ligand complexes could also trigger the following release of the entrapped proteins from 3D DNA.

### Protein@3D DNA-based paper sensor

**Fig. 5:**
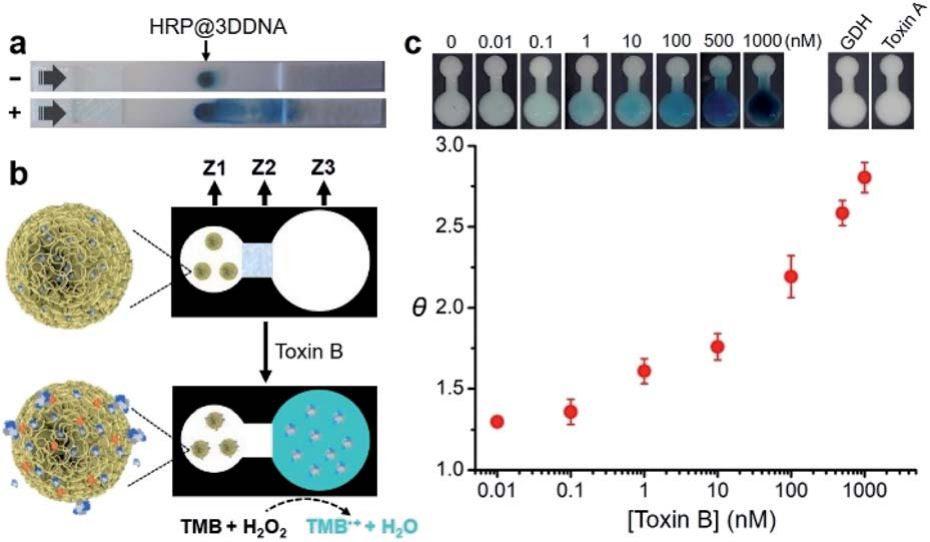
(a) Illustration of the release of HRP from immobilized HRP@3D DNA in response to toxin B on a paper strip. Lateral flow was first carried out in assay buffer without (−) and with (+) 100 nM toxin B, followed by the flow of reaction buffer containing TMB and H_2_O_2_. (b) An all-printed paper device and its working principle. **Z1**: sensing zone; **Z2**: valve zone; **Z3**: detection zone. (c) Sensitivity and selectivity of the paper device. *θ* = signal-to-background ratio.

Finally, we examined the possibility of exploiting fast-responding protein@3D DNA for biosensing applications. Previously, we have demonstrated that 3D DNAs can be easily printed onto paper to create functional paper sensors.^8*b*^ Taking advantage of this finding, we evaluated whether the particles could undergo on-command controlled release of proteins on paper. HRP@3D DNA particles incorporated with concatemeric aptamers (1.54 ± 0.23 μm in diameter, Fig. S17†) for toxin B (a biomarker for *Clostridium difficile*)^12^ were first synthesized by RCA, and deposited onto a lateral flow strip, composed of a sample pad, nitrocellulose membrane and an absorbent pad (Fig. 5a). After running the assay buffer through, followed by a reaction buffer containing chromogenic substrate TMB and H_2_O_2_, the particles remain immobilized at their initial locations and catalyze the oxidation of TMB to produce an insoluble blue colored product (TMB˙^+^). In the presence of toxin B, the loaded HRP molecules were indeed released, which have the ability to catalytically generate colored TMB˙^+^ to decorate the strip.

We then designed a simple colorimetric paper device that employs the HRP@3D DNA, as illustrated in Fig. 5b. It features three zones: a left sensing zone (**Z1**) that contains HRP@3D DNA (Fig. S18†), the middle valve zone (**Z2**) where the pullulan film (20%, wt/v) serves as a dissolvable bridge to control the flow rate of the fluid, and the right detection zone (**Z3**) where the substrate TMB was impregnated. These three zones are demarcated on a HF120 nitrocellulose membrane using wax barriers (Fig. S19†).

In a typical test, 15 μL of assay buffer containing toxin B was added to **Z1**. The pullulan film at **Z2** will be dissolved at around 15 min (Fig. S20†), thus allowing the flow of solution to **Z3**. Any released HRP will oxidize TMB in the presence of H_2_O_2_, generating a colorimetric readout at **Z3**.

The color intensity was found to be proportional to the concentration of toxin B (Fig. 5c). A limit of detection (LOD, defined as 3*σ*, *σ* = standard deviation of the blank samples) of 0.1 nM was achieved within 16 min. It is worth noting this device offers better sensitivity (LOD of 0.1 nM) and shorter sample-to-answer time (16 min) compared with our previous paper-based device using RCA (LOD of 0.6 nM, 40 min).^12^ We attribute this high detection sensitivity to the fact that a released HRP enzyme was able to generate a multitude of reporter molecules due to its high turn-over frequency. No obvious signal was observed when the device was tested with glutamate dehydrogenase (GDH), a general antigen of *Clostridium difficile* and toxin A (which shares 49% sequence identity to toxin B). Interestingly, we found that 3D DNA could increase the long-term stability of HRP. Native HRP stored in water lost 98% of its activity within two days at room temperature. In sharp contrast, HRP@3D DNA remained fully active for at least 50 days (Fig. S21†). Furthermore, the encapsulation of HRP into 3D DNA resulted in the retention of 49% of initial activity following a 5 minute heat treatment at 70 °C *versus* only 10% for native HRP (Fig. S22†). We also evaluated the biostability of HRP@3D DNA particles after treatment with cell lysates on paper. No obvious signal was observed at **Z3**, and SEM further confirmed the stability of HRP@3D DNA after the cell lysate treatment (Fig. S23†).

## Conclusions

In summary, we have demonstrated for the first time that RCA-derived DNA superstructures loaded with proteins, so-called protein@3D DNA, can be used as a stimuli-responsive gated material for controlled release of proteins in a fast-responsive manner. Guest proteins, such as HRP, rSPG, IgG and Cyt c could be encapsulated into 3D DNA with high loadings. We observe that the specific DNA–DNA interaction provides a general means to trigger the release with fast kinetics on the order of minutes, whereas the amount of released protein is highly dependent on the size and pI of protein. Higher release is generally obtained for small-sized protein with pI far below the pH of the reaction buffer. A further approach to release the loaded proteins involved the incorporation of concatemeric aptamers into the 3D DNA and the formation of aptamer–ligand complexes. Successful incorporation of two well-known DNA aptamers, one for a small molecule ATP and one for a protein PDGF, to HRP@3D DNA, demonstrates the generality of this approach.

We have also demonstrated that protein@3D DNA particles can be printed on paper device, allowing for rapid and colorimetric detection of *Clostridium difficile* toxin B. This device could be easily extended to other targets so long as the release process can be triggered by a specific aptamer–ligand recognition event. Given the fact that many aptamers against wide-ranging targets are available or can be obtained by *in vitro* selection,^10^ we envision that this gating system could play significant role in the development of new generations of controlled-delivery nanodevices and point-of-care testing devices for diverse applications in chemical biology, medical diagnostics, and biosensing.

## Author contributions

YZ and ML designed the study. YZ and QZ performed the experiments. FC, YC, and ML analyzed the data. YL made contribution to the discussions during the work. YZ and ML prepared the manuscript. All authors provided feedback on the paper.

## Conflicts of interest

There are no conflicts to declare.

## Supplementary Material

SC-012-D1SC00795E-s001
