# Supplementary material for: Fast-responding functional DNA superstructures for stimuli-triggered protein release
Source: Chem Sci. 2021 May 7;12(24):8282–7. doi: 10.1039/d1sc00795e (PMC8221054; doi:10.1039/d1sc00795e)
Supplement: SC-012-D1SC00795E-s001 [file SC-012-D1SC00795E-s001.pdf]

Electronic Supplementary Material (ESI) for Chemical Science.
This journal is © The Royal Society of Chemistry 2021

Section A: Experimental details

A1. Material and Instrumentation

The sequences of all DNA oligonucleotides are provided in Table S1. They were purchased from Shanghai Sangon Biological Engineering Technology & Services Co., Ltd. (Shanghai, China), and purified by 10% denaturing (8 M urea) polyacrylamide gel electrophoresis (dPAGE) or high-performance liquid chromatograph (HPLC). T4 polynucleotide kinase (PNK), T4 DNA ligase and HRP were purchased from Sangon Biotech. ϕ29 DNA polymerase (Polϕ29) *was* purchased from Dakewe Biotech Co., Ltd. (Beijing, China). 3,3’,5,5’-tetramethylbenzidine (TMB) was purchased from Beijing Solarbio Science and Technology Co., Ltd. H_2O_2 (30%) was purchased from Beijing HuaTeng. Human IgG, rSPG and Cyt c were purchased from Beijing Bersee Science and Technology Co., Ltd. SYBR™ Gold Nucleic Acid Gel Stain (10,000 × Concentrate in DMSO) was purchased from Thermo Fisher Scientific. All other chemicals were purchased from Sigma-Aldrich and used without further purification.

The UV-vis absorbance was monitored on a microplate reader (Tecan, Switzerland). The fluorescent images were obtained using a Typhoon 5 variable mode imager (GE Healthcare) and analyzed using Image Quant software (Molecular Dynamics). Fluorescence measurements were performed with a Cary Eclipse fluorescence spectrophotometer. TEM was performed on a JEOL 2010F. SEM was performed using a Hitachi UHR FE-SEM SU8010.

A2. General procedures

A2.1. Preparation of circular DNA template (Ct)

In total, 4 different Cts were used in this study and their sequences are provided in Table S1. Linear Ct precursor (300 pmol) was phosphorylated at 37 °C with 10 units (U) of PNK in 50 μL of 1 × PNK buffer (50 mM Tris-HCl, pH 7.6 at 25 °C, 10 mM $MgCl_2$, 5 mM DTT, 0.1 mM spermidine) with 2 mM ATP for 40 min. The reaction was stopped by heating at 90 °C for 3 min. Upon cooling to room temperature (RT), the mixture was first mixed with DNA primer (Dp, 400 pmol) in 98 μL of 1 × T4 DNA ligase buffer (40 mM Tris-HCl, 10 mM $MgCl_2$, 10 mM DTT, 0.5 mM ATP, pH 7.8 at 25 °C), heated to 90 °C for 2 min, and then cooled at RT for 15 min. 10 U of T4 DNA ligase was then added (total volume: 100 μL). After incubation at RT for 2 hours, the obtained Cts were concentrated by standard ethanol precipitation and purified by 10% denaturing PAGE (dPAGE, 8 M urea).

A2.2. Synthesis of protein@3D DNA

In a typical experiment, 5 μL of 10 × rolling circle amplification (RCA) buffer

(500 mM Tris-HCl, 100 mM $(NH_4)_2SO_4$, 40 mM DTT, 100 mM $MgCl_2$, pH 7.5), 1 μL of Polϕ29 (10 U/μL), 8 μL of 2.5 mM dNTPs, 2 μL of 5 μM Ct (*e.g.,* Ct1, Ct2, Ct3, or Ct4), 2 μL of 10 μM Dp (*e.g.,* Dp1, Dp2, Dp3, or Dp4), 10 μL of 50 μM selected protein (*e.g.,* HRP, rSPG, IgG, or Cyt c), and 22 μL of ddH_2O (total volume: 50 μL) were incubated at 30 °C for 30 min before heating at 65 °C for 10 min to deactivate Polϕ29. The resulting mixture was incubated at RT for 12 h. The obtained protein@3D DNA particles were washed with ddH_2O three times by centrifugation at 14000 rpm for 10 min, and then re-dispersed in ddH_2O.

A3. Specific experiments described in the main manuscript

A3.1. SEM and TEM images of protein@3D DNA (Fig. 2a and 2b)

SEM samples were prepared by solvent evaporation on a clean silicon wafer or paper. The as-prepared samples were first coated with platinum using Sputter Coater before SEM imaging. TEM samples were prepared by solvent evaporation on a carbon grid.

A3.2. CFM images of F-HRP@3D DNA (Fig. 2c)

F-HRP@3D DNA was prepared using the general protocol described in **A2.2** except for: FITC-labeled HRP was used. CFM imaging was performed at RT using the Olympus IX83 inverted microscope (40 × objective). CFM samples were prepared by solvent evaporation on a glass.

A3.3. Quantification of the total number of F-HRP per F-HRP@3D DNA particle (Fig. 2d)

Calculation of the number of F-HRP@3D DNA particles. F-HRP@3D DNA particles with different amounts of F-HRP were prepared using the general protocol described in **A2.2** except for: 10 μL of F-HRP with various concentrations (25, 37.5, 50, 75, 100, and 125 μM) was used. 1 μL of F-HRP@3D DNA (dissolved in 50 μL) was added to 199 μL of 50 mM Tris-HCl buffer solution containing 1 × SYBR Gold (pH 7.5) and incubated at RT for 10 min. 10 μL of the mixture was then added to a hemocytometer and incubated at RT for 10 min. Bright field and fluorescent images of 80 squares on the hemocytometer were taken using the Olympus IX83 inverted microscope (Olympus, Japan) equipped with a 40-fold objective. The obtained images were analyzed with an ImageJ software to calculate the number of particles. The total number of F-HRP@3D DNA particles (N) in 1 μL of sample was calculated using: $N = [n/(1/4000)\ \mu L] \times 10\ \mu L \times 20$, where n is the average number of particles observed in each square, 1/4000 is the volume of each square, and 20 is the volume correction factor.

Calculation of the number of loaded F-HRP in 3D DNA.

50 μL of F-HRP@3D DNA was placed in the cuvette and the fluorescence intensity at 525 nm was recorded. Quantification of the loaded HRP was achieved by comparing the fluorescence intensity values of the test samples

to a standard curve. The maximum number of loaded F-HRP *($N_{F\text{-}HRP}$)* in 50 µL of F-HRP@3D DNA was determined using: $N_{F\text{-}HRP}$ = 0.08 µM × 6.02 × 10^{23} × 10^{-6} × (50 × 10^{-6}) L = 2.4 × 10^{12}, where 0.08 µM is the calculated maximum concentration of F-HRP loaded into 3D DNA.

Calculation of the maximum number of loaded F-HRP per particle. The maximum number of loaded F-HRP for each particle ($N_{F\text{-}HRP}$*/particle*) was determined using $N_{F\text{-}HRP}$*/particle* = $N_{F\text{-}HRP}$ / N = 2.4 × 10^{12}/{[1.4/(1/4000) µL] × 10 µL × 20 × 50]} = 4.3 × 10^{4}, where 1.4 is the average number of particles observed in each square.

A3.4. Determination of total number of MA molecules per HRP@3D DNA particle (Fig. 2d inset)

Each Ct was encoded with an *Eco*RV recognition site (*GATATC*). First, 1 µL of HRP@3D DNA with different dilution times (× 15, × 20) was mixed with 4 µL of digestion template (*e.g.,* Dt1, Dt2, or Dt3), 2 µL of 10 × *Eco*RV buffer and 10 µL of water, heated at 90 °C for 5 min before cooling to RT for 15 min. 3 µL of *Eco*RV (15 U/µL) was then added, and the mixture was incubated at 37°C for 24 h. 1 µL of 1 µM internal control (IC1, 59 nt) and 21 µL of 2 × urea PAGE loading buffer were then added. The mixture was then run on a 10% dPAGE gel and stained with 1 × SYBR Gold at 4°C for 10 min before scanning. Image Quant software was used to estimate the fluorescence intensity of monomeric DNA bands ($F_{51\ nt}$) and IC band ($F_{59\ nt}$) The total number of MA molecules per particle (N_{MA}*/particle*) can be calculated using: N_{MA}*/particle* = [($F_{51\ nt}/F_{59\ nt}$) × 1 pmol × 20 × 10^{-12} × (6.02 × 10^{23})] / (1.12 × 10^{6}), where 1.12×10^{6} is the total number of particles in 1 µL of sample, 20 is the volume correction factor.

A3.5. Kinetic release of HRP or F-HRP from 3D DNA (Fig. 3b)

10 µL of HRP@3D DNA or F-HRP@3D DNA was first mixed with 5 µL of 10 µM ^{PM}DO-22, 30 µL of ddH_2O and 5 µL of 10 × PBS buffer (containing 50 mM $MgCl_2$, pH 7.4) (total volume: 50 µL). After vortexing for 5 s, the mixture was incubated at RT for 0.5, 1, 5, 10, 20, 30 min and centrifuged at 14000 rpm for 1 min. Photographs of the collected F-HRP@3D DNA at 20 min were captured by a smartphone camera or gel imaging system (Tanon 3500R). The supernatant was then collected. The concentration of free HRP in the supernatant was calculated by determining its initial catalytic rate in the presence of TMB and H_2O_2. In brief, 42.5 µL of HOAc-NaOAc buffer (0.1 M, pH 4.5) was first mixed with 50 µL of the above-collected supernatant and 2.5 µL of 20 mM TMB in 96-well plates. 5 µL of 100 mM H_2O_2 was then added before recording the UV-vis absorption at 652 nm. Quantification of HRP was achieved by comparing the initial velocity values of the test samples to a standard curve.

A3.6. ^{PM}DO-22 concentration-dependent release of HRP (Fig. 3c)

10 μL of HRP@3D DNA (~ 10^7 particles), 5 μL of 10 × PBS buffer (containing 50 mM $MgCl_2$, pH 7.4), 5 μL of ^{PM}DO-22 (0.5, 1, 2, 4, 6, 10, 20, 40, 60, and 80 μM) and 30 μL of ddH_2O (total volume: 50 μL) were incubated at RT for 20 min. This was followed by centrifugation at 14000 rpm for 1 min. The supernatant was then collected to calculate the amount of free HRP according to the protocols described in **3.5**.

A3.7. DO length-dependent release of HRP or F-HRP (Fig. 3d)
10 μL of HRP@3D DNA or F-HRP@3D DNA, 5 μL of 10 × PBS buffer (containing 50 mM $MgCl_2$, pH 7.4), 5 μL of 20 μM DO (*e.g.,* ^{PM}DO-10, ^{PM}DO-22a, ^{PM}DO-30, or ^{PM}DO-40) and 30 μL of ddH_2O (total volume: 50 μL) were incubated at RT for 20 min. This was followed by centrifugation at 14000 rpm for 1 min before imaging using Gel imaging system (Tanon 3500R). The supernatant was then collected to calculate the released HRP according to the protocols described in **3.5**.

A3.8. DO sequence-dependent release of HRP or F-HRP (Fig. 3e)
The relevant experiments were done very similarly to the protocols described in **A3.7** except for: the use of 6 μM DOs including ^{PM}DO-22a, 1MM A/A, 1MM C/C, 3MM, and polyT_{20}.

A3.9. ATP concentration-dependent release of HRP (Fig. 4c)
HRP@3D DNA containing concatemeric ATP aptamers was prepared using the protocol described in **A2.2** except for: the use of Ct2 and Dp2. The protocols used for the release experiments are very similar to the protocols described in **A3.6** except for: (1) different concentrations of ATP (final concentration: 0.1, 1, 2, 2.5, 4, 5, 8, and 10 mM) were tested; (2) ATP assay buffer (20 mM Tris-HCl, 100 mM NaCl, 5 mM $MgCl_2$, pH 7.8) was used. To test the selectivity, GTP with a final concentration of 5 mM was used.

A3.10. PDGF concentration-dependent release of HRP (Fig. 4d)
HRP@3D DNA containing concatemeric PDGF aptamers was prepared using the protocol described in **A2.2** except for: the use of Ct3 and Dp3. The protocols used for the release experiments are very similar to the protocols described in **A3.6** except for: (1) different concentrations of PDGF (final concentration: 50, 100, 200, 300, 400, 500, 750, 1000 nM) were tested; (2) PDGF assay buffer (20 mM Tris-HCl, 100 mM NaCl, 5 mM KCl, 5 mM $MgCl_2$, pH 7.8) was used. To test the selectivity, BSA with a final concentration of 1 μM was used.

A3.11. Release of HRP from HRP@3D DNA on lateral flow strip (Fig. 5a)
HRP@3D DNA was first prepared according to the one described in **A2.2** except for: the use of Ct4 and Dp4. For the fabrication of lateral flow strip, sample pads were prepared by cutting Millipore C083 cellulose sheets

(GFCP103000, Millipore) into 0.5 cm × 1 cm strips. The strips were treated with 0.5% v/v Tween 20 and 0.5% w/v BSA for 30 minutes, then dried at RT. Absorbent pads (AP1002500, Millipore) were cut into 0.5 cm × 2 cm strips. The nitrocellulose membrane (Millipore HF180) was cut into a 3 cm × 0.5 cm strip. 1 μL of F-HRP@3D DNA was dropped onto the strip and air dried. The strips were treated with 0.5% w/v BSA for 10 minutes, then dried at RT. The entire test strip was assembled on a 0.5 cm × 6 cm DCM backing card. To test the release of HRP from HRP@3D DNA on paper, 100 μL of assay buffer (20 mM Tris-HCl, 100 mM NaCl, 5 mM KCl and 10 mM $MgCl_2$, 1 mM DTT, 0.05% Tween-20, pH 7.5) containing 100 nM toxin B was added to the sample pad. After 20 min, 100 μL of reaction buffer (1 × PBS) containing freshly prepared TMB (final concentration: 4 mM) and H_2O_2 (final concentration: 2 mM) was then added. The strip was then imaged with a Galaxy Note10 mobile phone camera.

A3.12. Fabrication of paper device.

The paper device design was drawn using Microsoft PowerPoint. Wax was printed using a Xerox ColorQube 8570N solid wax printer followed by heating at 120°C for 2 min. This produced a patterned paper with a sensing zone (**Z1**, 0.4 cm diameter), a middle valve zone (**Z2**, 0.2 cm long and 0.2 cm wide), and a detection zone (**Z3**, 0.6 cm diameter). An optimized volume (~ 5 μL) of the HRP@3D DNA complex was then printed onto **Z1** using a Biodot XYZ3060 automated dispensing unit. After immersion into the blocking buffer (25 mM PBS containing 1% BSA, 0.02% Tween-20, pH 7.5) for 20 min, the obtained bioactive paper was dried at RT. 500 μL of 20% (w/v) pullulan in 1 × PBS buffer was prepared. 1.5 μL of this solution was dispensed onto **Z2** and air dried to form a transparent film. Next, 5 μL of 20 mM TMB (dissolved in DMSO) was dispensed onto **Z3**, air dried and stored at RT until use.

A3.13. Sensitivity and selectivity test of the paper device (Fig. 5c).

15 μL of assay buffer (20 mM Tris-HCl, 100 mM NaCl, 5 mM KCl and 10 mM $MgCl_2$, 1 mM DTT, 0.05% Tween-20, pH 7.5) containing toxin B (0.01-1000 nM) was applied to the **Z1**. Following incubation for 15 min, the pullulan film at **Z2** was dissolved, and the buffer was allowed to migrate to the **Z3**. Then 10 μL of HOAc-NaOAc buffer (0.1 M, pH 4.5) containing 2 mM H_2O_2 was applied to the **Z3**. After 1 min, the produced color was captured using a a Galaxy Note10 mobile phone camera and analyzed using ImageJ software. For the selectivity test, the protocol is similar to the one described above except for: 100 nM of GDH and toxin A were used.

Section B. Supplementary Tables and Figures.

Table S1. Sequences of DNA oligonucleotides used in this work. The sequence portions in bold is the complementary sequence for ATP aptamer, PDGF aptamer and toxin B aptamer, respectively.

DNA oligonucleotide	Sequence (5′-3′)
**Precursor of circular DNA template (Ct)**	
Linear Ct (Ct1, 51 nt)	ATTCGTGTGA GAAAACCCAA CCCGCCCTAC CCAAAAGATA TCGTCAGATG A
Linear ATP aptamer Ct (Ct2, 69 nt)	TGTCTTCGCC GATATCTATA GTGA**ACCTTC CTCCGCAATA CTCCCCCAGG T**ATCTTTCGA CTAAGCACC
Linear PDGF aptamer Ct (Ct3, 67 nt)	TGCAGCGAGA TATCCTCA**CA GGATCATGGT GATGCTCTAC GTGCCGTAGC CTG**CCCTTTC GACTACC
Linear toxin B aptamer Ct (Ct4, 70 nt)	ATATAGTAGA AACCACTATA T**AGTCTGACT AACACATCGC ACCAAGCACC GAACCAGACT** CTTCGCCGTC
**DNA primer (Dp) for RCA**	
Dp1 for Ct1 (20 nt)	CTCACACGAA TTCATCTGAC
Dp2 for Ct2 (21 nt)	GGCGAAGACA GGTGCTTAGT C
Dp3 for Ct3 (22 nt)	ATCTCGCTGC AGGTAGTCGA AA
Dp4 for Ct4 (20 nt)	TCTACTATAT GACGGCGAAG
**DNA template (Dt) for digestion**	
Dt1 (22 nt)	CCCAAAAGAT ATCGTCAGAT GA
Dt2 (24 nt)	GTCTTCGCCG ATATCTATAG TGAA
Dt3 (24 nt)	TGCAGCGAGA TATCCTCACA GGAT
**DNA oligonucleotides for study of release**	
^{PM}DO-22a (22 nt)	CCCAAAAGAT ATCGTCAGAT GA
^{PM}DO-22b (22 nt)	TGAGAAAACC CAACCCGCCC TA
1 MM A/A (22 nt)	CCCAAAAGAT A<u>A</u>CGTCAGAT GA
1 MM C/C (22 nt)	CCCAAAA<u>C</u>AT ATCGTCAGAT GA
3 MM (22 nt)	CCCAAAA<u>C</u>AT ATC<u>C</u>TCAGAT <u>C</u>A
poly T_{20} (20 nt)	TTTTTTTTTT TTTTTTTTTT
^{PM}DO-10 (10 nt)	CCCAAAAGAT
^{PM}DO-30 (30 nt)	CCCAAAAGAT ATCGTCAGAT GAATTCGTGT
^{PM}DO-40 (40 nt)	CCCAAAAGAT ATCGTCAGAT GAATTCGTGT GAGAAAACCC
**5’-amino-modified DNA oligonucleotide**	
**H_2N-DNA (30 nt)**	NH_2-TTTTTTTTTT GTCAGATGAA TTCGTGTGAG
**DNA internal control (IC)**	
IC1 (59 nt)	TGTCTTCGCC GATAACCTTC CTCCGCAATA CTCCCCCAGG TATCTTTCGA CTAAGCACC
IC2 (77 nt)	ATCGAACCAG GTCGGGGCCG AAATATAGGA TATTTTGGGA GGCTATGCTA GGTAGCTTAT

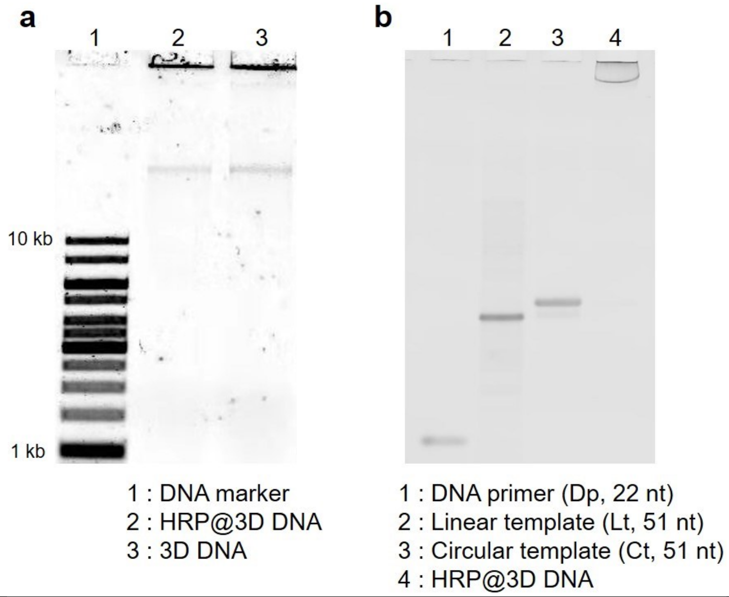

CAGACTGATG TTGAGAT

Fig. S1. (a) 0.6% agarose gel and (b) 10% denaturing PAGE (dPAGE) analysis of 3D DNA and HRP@3D DNA.

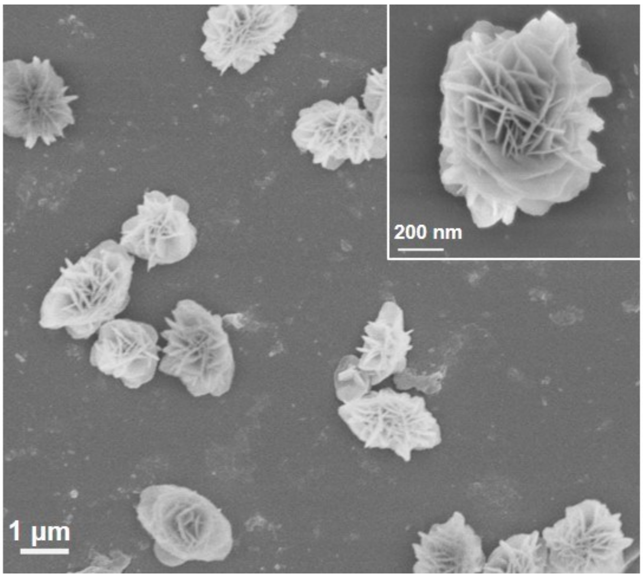

Fig. S2. SEM images of 3D DNA particles. These particles were prepared according to the one described in **A2.2** except for: HRP was not included.

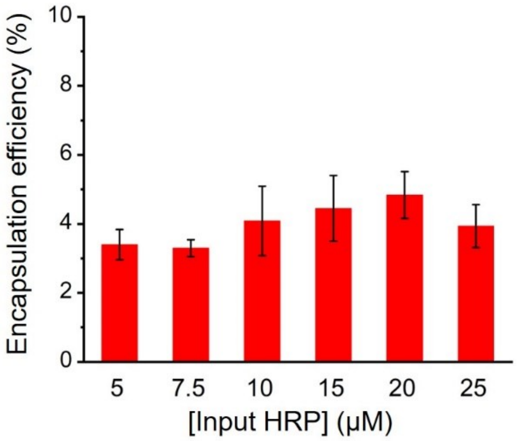

Fig. S3. Input HRP concentrations vs. encapsulation efficiency.

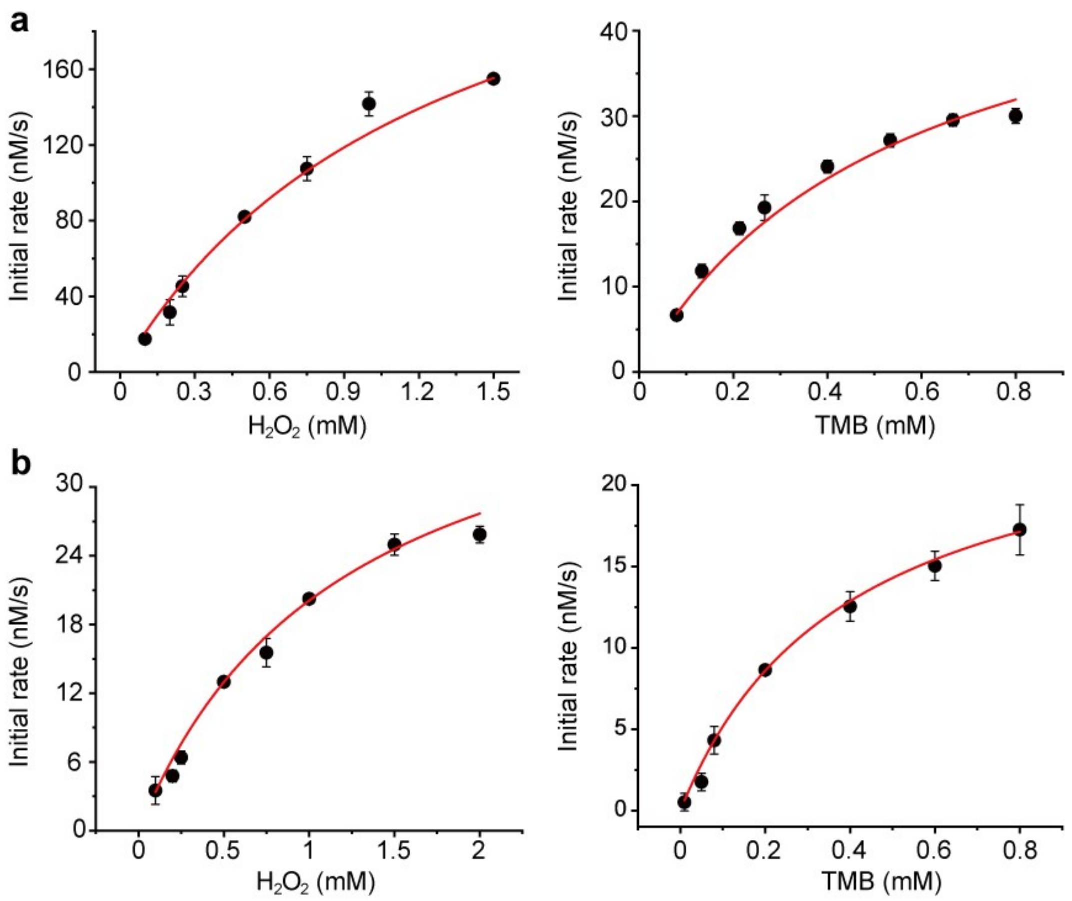

Fig. S4. Michaelis-Menten plot of (a) native HRP compared with that of (b) HRP@3D DNA using H_2O_2 and TMB as substrates.

Experimental details: *For the kinetic assay using H_2O_2 and varying concentrations of TMB:* 84 μL of 0.1 M HOAc-NaOAc buffer (pH 4.5), 1 μL of 40 nM native HRP or 80 nM HRP@3D DNA, 10 μL of 100 mM H_2O_2, and 5 μL of different concentrations of TMB (final concentration: 0.01-0.8 mM) were added into plastic 96-well plate, and incubated at RT for 20 min in the dark. Absorbance values at 652 nm were monitored in real-time on the Tecan Spark reader (for the final product TMB radical cation, ε_{652nm} = 3.9 × 10^4 M^{-1} cm^{-1}). The Michaelis-Menten constant (K_m) and maximum velocity (V_{max}) were determined by fitting Michaelis-Menten equation using Origin software: $V = V_{max} \cdot [S] / (K_m + [S])$, where $[S]$ was the substrate concentration. The turnover number (k_{cat}) was calculated using $k_{cat} = V_{max}/[E]$, where $[E]$ is the concentration of native HRP or HRP@3D DNA.

For the kinetic assay using TMB and varying concentrations of H_2O_2: The relevant experiment was performed in the same way as described above except that: 5 μL of 0.6 mM TMB and 10 μL of different concentrations of H_2O_2 (final concentration: 0.1-2 mM) were used.

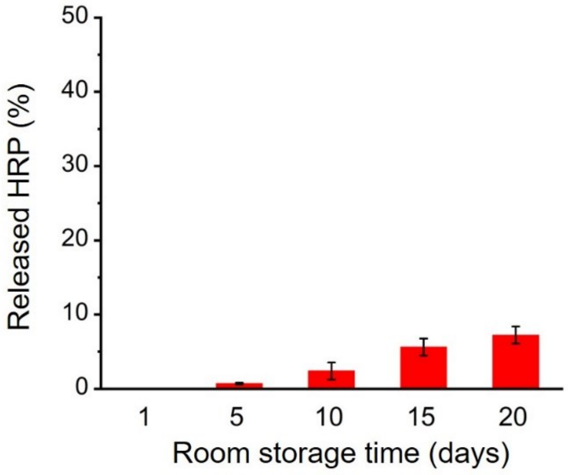

Fig. S5. Relative released HRP (%) during room-temperature storage in the absence of [PM]DO-22a.

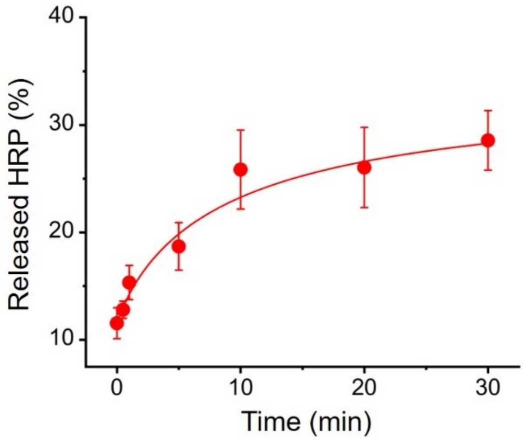

Fig. S6. Kinetic release of 3D DNA-adsorbed HRP particles in the presence of 1 μM ^{PM}DO-22a.

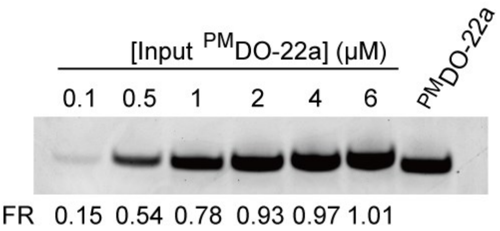

Fig. S7. dPAGE analysis of the bound ^{PM}DO-22a.

Experimental details: _dPAGE analysis of the bound ^{PM}DO-22a:_ 10 μL of HRP@3D DNA, 5 μL of 10 × PBS buffer (containing 50 mM $MgCl_2$), 5 μL of ^{PM}DO-22a (final concentration: 0.1, 0.5, 1, 2, 3, and 4 μM) and 30 μL of ddH_2O (total volume: 50 μL) were incubated at RT for 10 min. This was followed by centrifugation at 14000 rpm for 1 min. After washed by 50 μL of 1 × PBS buffer for three times to remove the unbound ^{PM}DO-22a, the obtained precipitation was dissolved in 100 μL of ddH_2O. 10 μL of the above solution was first mixed with 10 μL of 2 × urea PAGE loading buffer before heated at 90 °C for 10 min. The mixture (total volume: 20 μL) and 20 μL of 0.05 μM ^{PM}DO-22a were run on a 10% dPAGE gel. After stained with 1×SYBR Gold at 4 °C for 10 min, the gel was scanned and analyzed by Image Quant software.
Calculation of the number of bound ^{PM}DO-22a: The fluorescence intensity of bound ^{PM}DO-22a band ($F^{PM}{}_{DO\text{-}22a}$) and the IC band (F_{IC}) was estimated using Image Quant software. The fluorescence ratio (FR) of the two bands was then determined. The amount of bound ^{PM}DO-22a ($N^{PM}{}_{DO\text{-}22a}$) at [input ^{PM}DO-22a] = 6 μM was then calculated using: $N^{PM}{}_{DO\text{-}22a}$ = 1.01 × 1 pmol × 10^{-12} × 6.02 × 10^{23} × 10 = 6.08 × 10^{12}, where 10 is the volume correction factor.
Calculation of the number of released HRP per DNA binding event: The amount of loaded HRP in 10 μL of HRP@3D DNA was determined to be 4.8 × 10^{11} according to the protocol described in **A3.3**. The total released HRP (%) at [input ^{PM}DO-22a] = 6 μM was calculated to be (80.8 ± 5.7) %. The number of released HRP (N_{HRP}) from 3D DNA was then determined using: N_{HRP} = 80.8% × 4.8 × 10^{11} = 3.87 × 10^{11}. Thus the number of released HRP per DNA binding event ($N_{HRP}/^{PM}DO\text{-}22a$) was calculated using: $N_{HRP}/^{PM}DO\text{-}22a$ = 3.87 × 10^{11} / 6.08 × 10^{12} = 1/15.

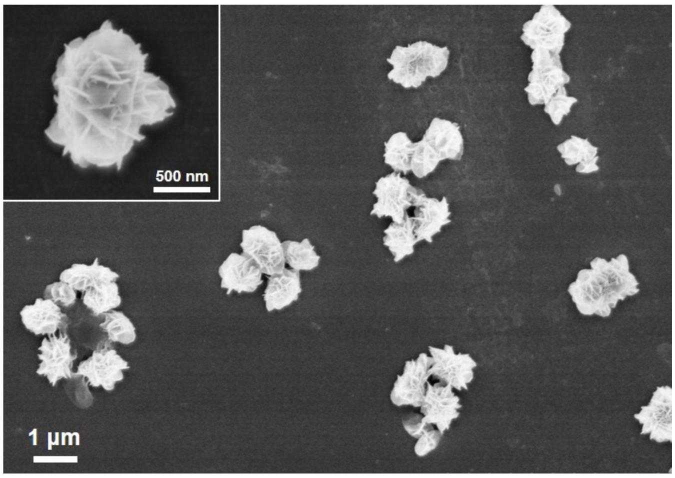

Fig. S8. SEM images of HRP@3D DNA after DNA hybridization. [^{PM}DO-22a] = 1 μM.

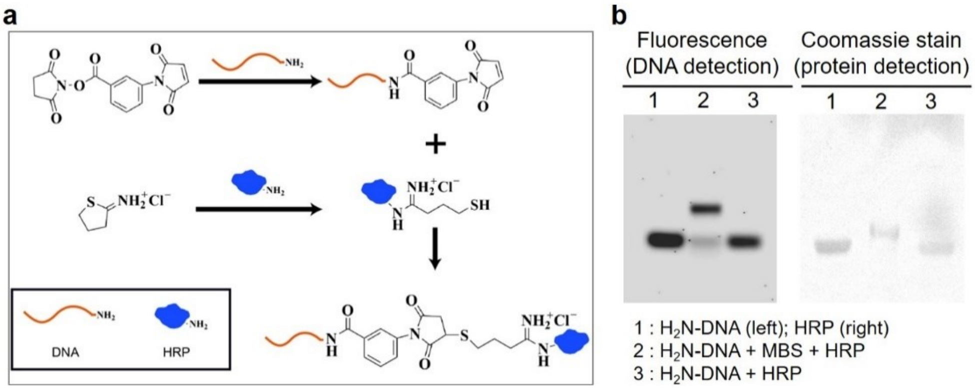

Fig. S9. (a) Conjugation of H_2N-DNA to HRP using MBS and 2-Iminothiolane. (b) Analysis of H_2N-DNA-HRP conjugation mixtures using agarose gel electrophoresis and SDS-PAGE.

Experimental details: (1) 3.2 μL of 6.4 mM MBS (dissolved in DMSO) was mixed with 10 μL of 100 μM H_2N-DNA and adjusted to a final reaction volume of 100 μL with 1 × PBS buffer. The mixture was incubated at RT for 1 h, and passed through a membrane based molecular sizing centrifugal column with a molecular weight cut-off of 3 kDa (NANOSEP OMEGA, Pall Incorporation) to remove the excess MBS. The column was washed 3 times with 50 μL of 1 × PBS buffer and the activated H_2N-DNA was resuspended in 50 μL of 1 × PBS buffer (denoted as **Product A**).

(2) 10 μL of 50 μM HRP was mixed with 10 μL of 600 μM 2-Iminothiolane and adjusted to a final reaction volume of 100 μL with 1 × PBS buffer. After incubated at RT for 30 min, the mixture was filtered through 3 kDa cut-off centrifugal column. The column was washed 3 times with 50 μL of 1 × PBS buffer, and the activated HRP was resuspended in 50 μL of 1 × PBS buffer (denoted as **Product B**).

(3) **Product A** was mixed with **Product B,** and incubated at RT for 2 h. The mixture was filtered through 30 kDa cut-off centrifugal column. The H_2N-DNA-HRP conjugate was then washed 3 times with 100 μL of 1 × PBS buffer and re-suspended with 50 μL of 1 × PBS buffer before analyzed by agarose gel electrophoresis and SDS-PAGE.

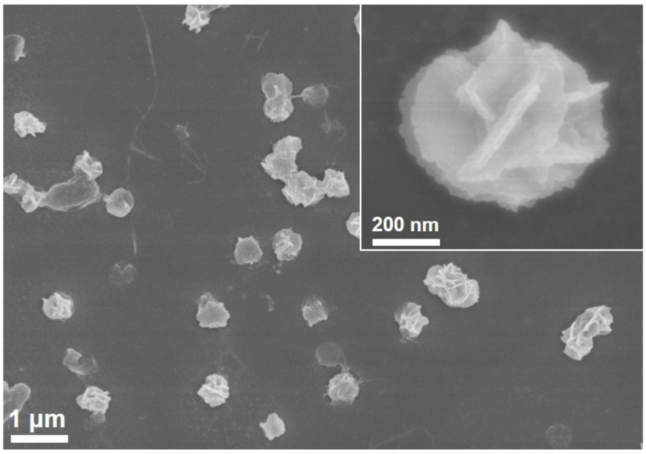

Fig. S10. SEM images of H_2N-DNA-HRP@3D DNA particles. These particles were prepared according to the one described in **A2.2** except for: H_2N-DNA-HRP was used.

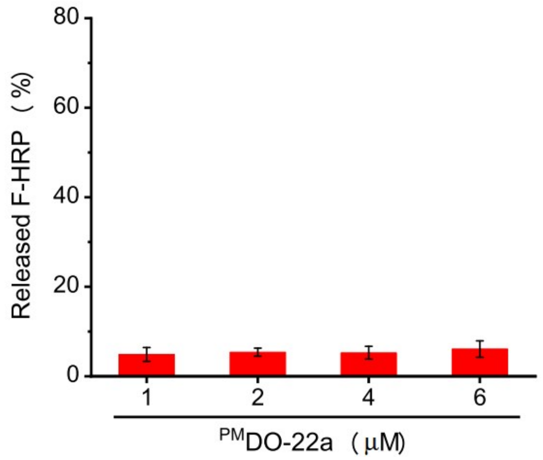

Fig. S11. Relative released HRP (%) from H_2N-DNA-HRP@3D DNA in the presence of different concentrations of ^{PM}DO-22a.

Experimental details: The protocol is similar to the one described in **A3.6** except for: H_2N-DNA-HRP@3D DNA was used.

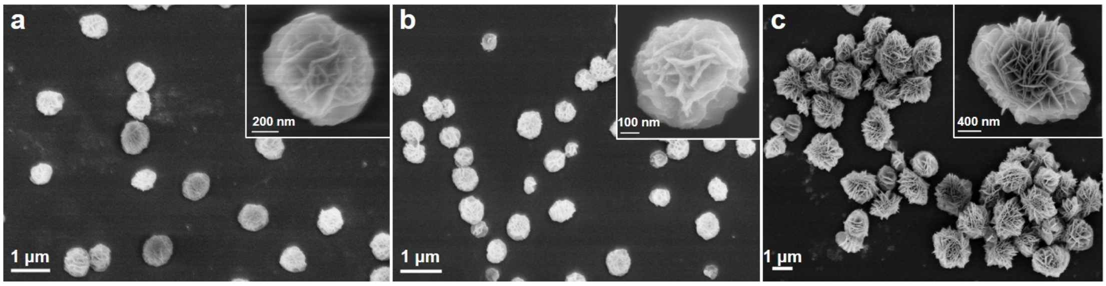

Fig. S12. SEM images of (a) Cyt c@3D DNA (0.56 ± 0.09 μm in diameter), (b) rSPG@3D DNA (0.68 ± 0.17 μm in diameter), (c) IgG@3D DNA (1.69 ± 0.17 μm in diameter). These particles were prepared according to the one described in **A2.2** except for: Cyt c, rSPG, or IgG was used.

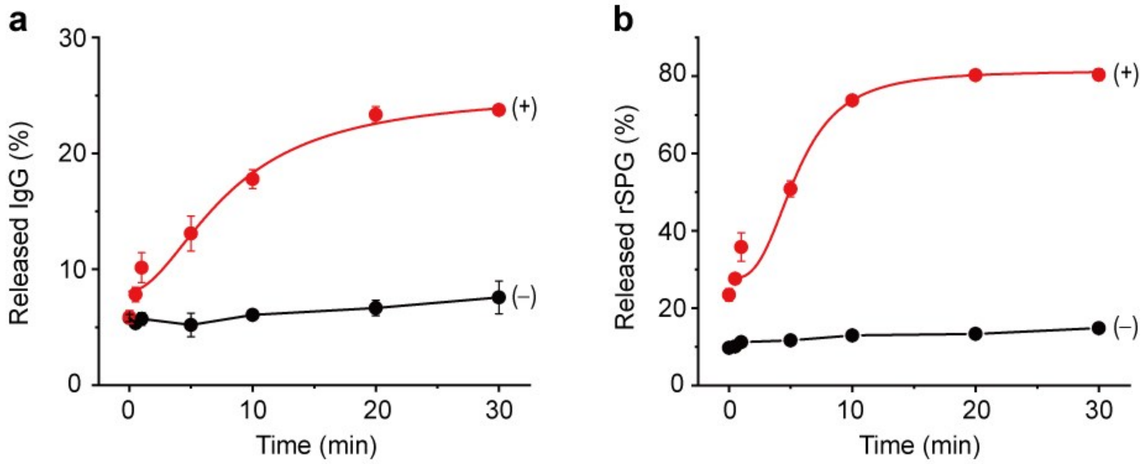

Fig. S13. Kinetic release of (a) IgG and (b) rSPG from 3D DNA in the absence (-) and presence (+) of 1 μM ^{PM}DO-22a in 1 × PBS buffer (containing 5 mM $MgCl_2$, pH 7.4).

Experimental details: The protocol is similar to the one described in **A3.5** except for: 1) FITC-labeled rSPG@3D DNA and FITC-labeled IgG@3D DNA were used. 2) Quantification of the release proteins was achieved by comparing the fluorescence intensity values of the test samples to a standard curve.

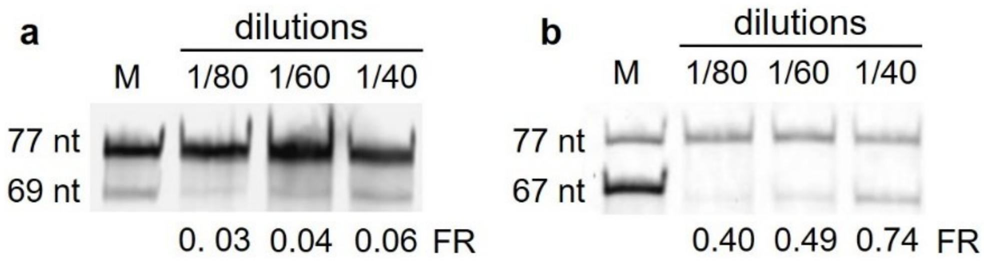

Fig. S14. dPAGE analysis of digested HRP@3D DNA containing (a) concatemeric ATP aptamers and (b) concatemeric PDGF aptamers. Top band: DNA internal control (IC2, 77 nt). Bottom band: monomeric ATP aptamers (69 nt) or PDGF aptamers (67 nt). FR: ratio of fluorescence intensity of the 69 nt (or 67 nt) and 77 nt DNA bands.

Experimental details: The protocol is similar to the one described in **A3.4** except for: 1) Dt2 and Dt3 were used for the digestion of HRP@3D DNA containing concatemeric ATP aptamers and PDGF aptamers, respectively.

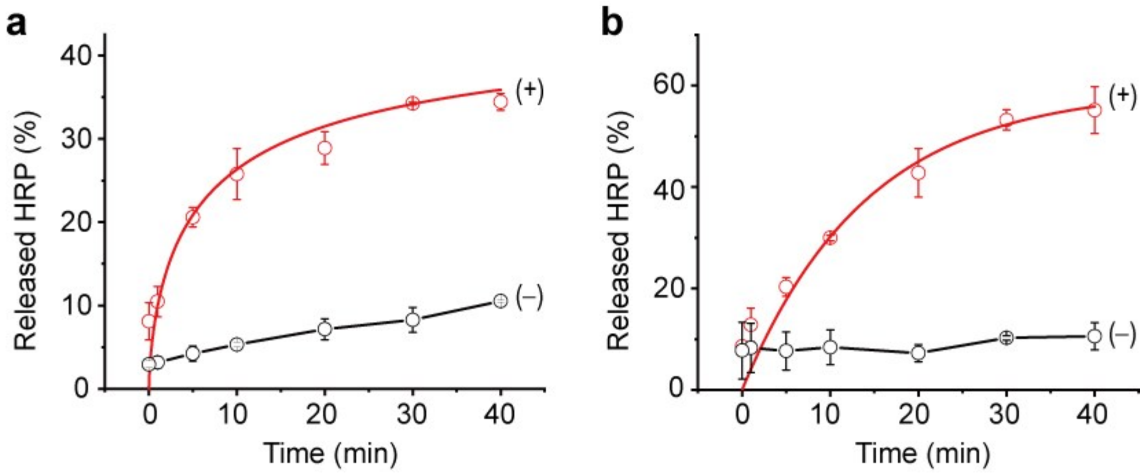

Fig. S15. Kinetic release of HRP from 3D DNA in the absence (-) and presence (+) of (a) 5 mM ATP and (b) 0.4 μM PDGF.

Experimental details: The protocol is similar to the one described in **A3.5** except for: 1) 5 mM ATP and 0.4 μM PDGF, and 2) ATP assay buffer (20 mM Tris-HCl, 100 mM NaCl, 5 mM $MgCl_2$, pH 7.8) and PDGF assay buffer (20 mM Tris-HCl, 100 mM NaCl, 5 mM KCl, 5 mM $MgCl_2$, pH 7.8) were used.

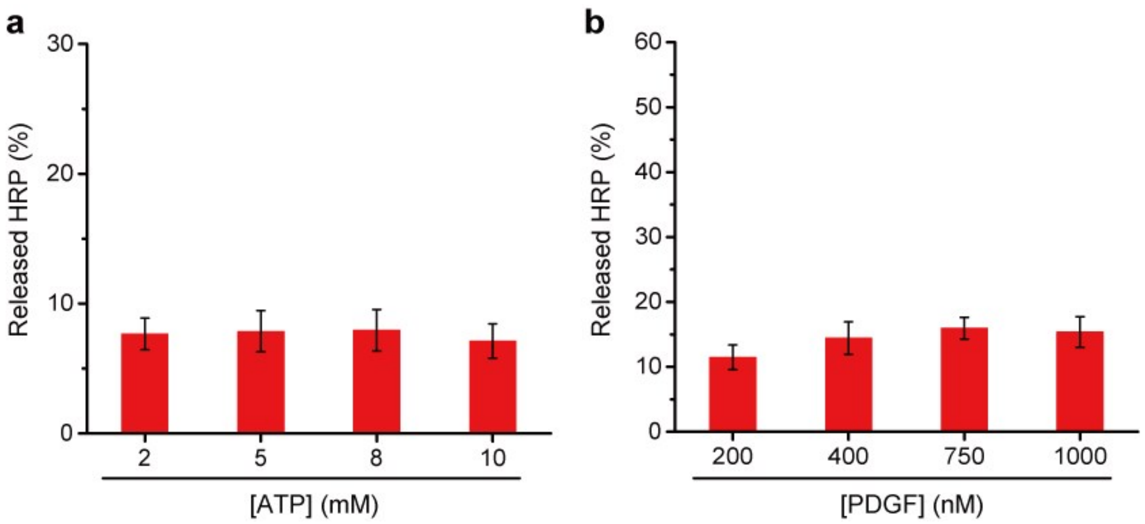

Fig. S16. Relative released HRP (%) from HRP@3D DNA containing mutant aptamer sequence in the presence of different concentrations of (a) ATP and (b) PDGF.

Experimental details: The protocol is similar to the one described in **A3.9 and A3.10** except for: HRP@3D DNA was made from Ct1 and Dp1 according to the protocols described in **A2.2**.

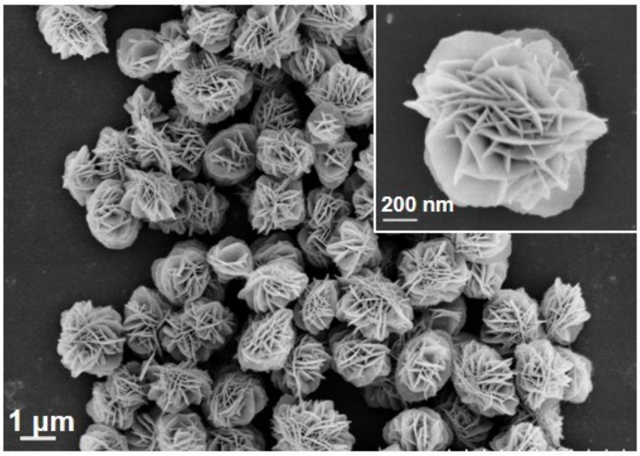

Fig. S17. SEM images of HRP@3D DNA incorporated with concatemeric aptamers for toxin B. HRP@3D DNA was prepared according to the one described in **A2.2** except for: Ct4 and Dp4 were used.

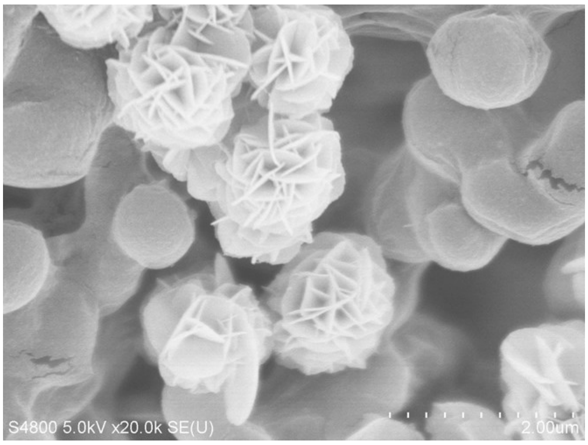

Fig. S18. SEM images of HRP@3D DNA at **Z1**.

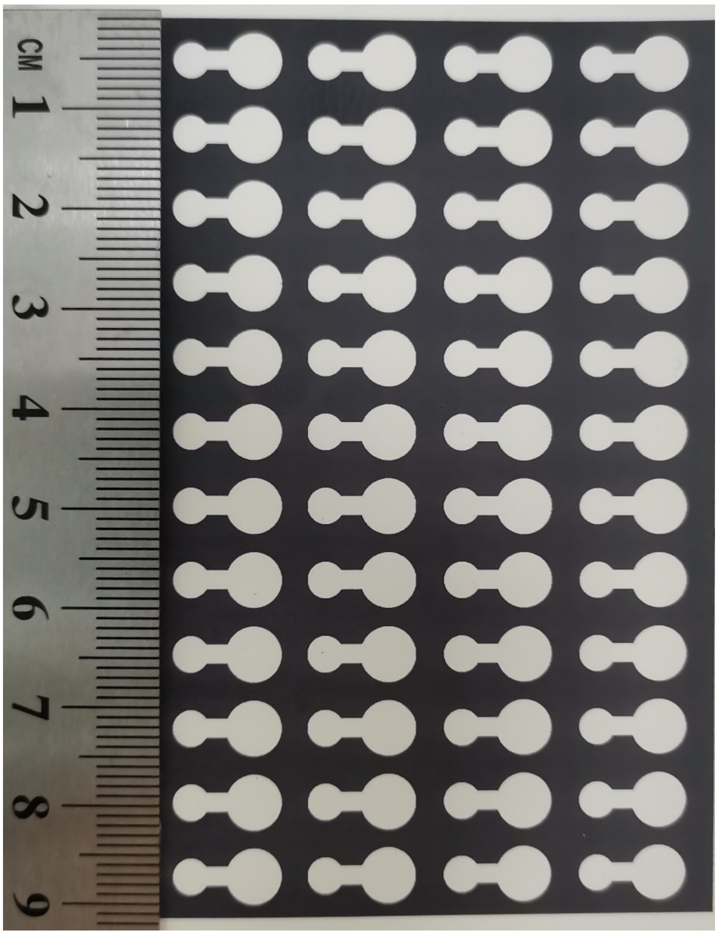

Fig. S19. Paper printing samples. Black part indicates wax barrier and white portion is the device with different zones including **Z1**, **Z2** and **Z3**. The experimental details were described in **A3.12**.

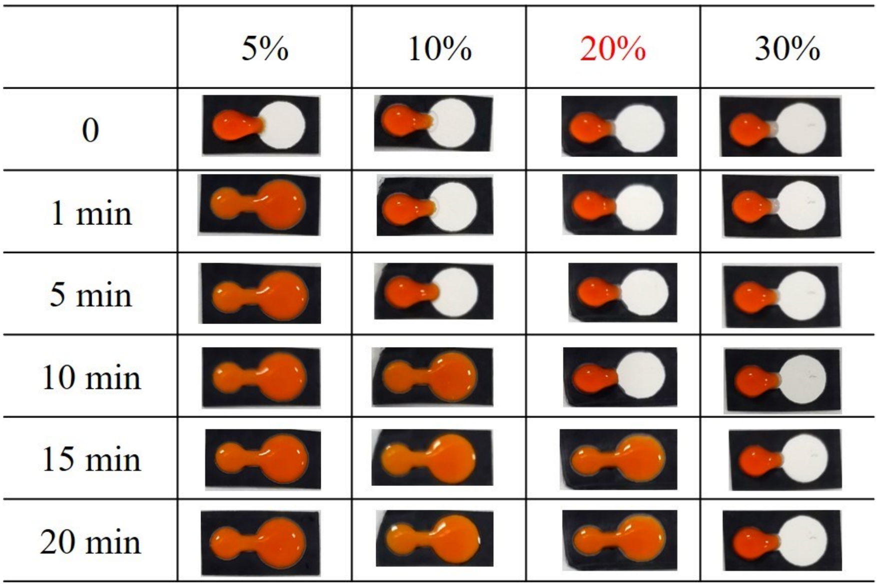

Fig. S20. Flow of 15 μL of dye solutions (10 mM Tris-HCl, 0.01% Methyl orange, pH 7.5) through paper device containing dried pullulan films (5%, 10%, 20%, and 30%) at **Z2**.

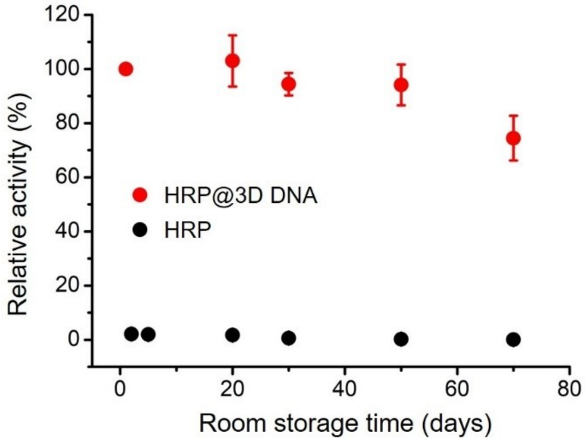

Fig. S21. Evaluation of the long-term stability of HRP@3D DNA and native HRP stored at RT.

Experimental details: 5 nM HRP and 2 μL of HRP@3D DNA in 50 μL of ddH_2O were stored at RT for different periods of time. The above solution was mixed with 50 μL of HOAc-NaOAc buffer (0.1 M, pH 4.5) and 5 μL of 20 mM TMB in 96-well plates. 5 μL of 100 mM H_2O_2 was then added before recording the UV-vis absorption at 652 nm. Quantification of the activity was achieved by comparing the initial velocity values of the test samples.

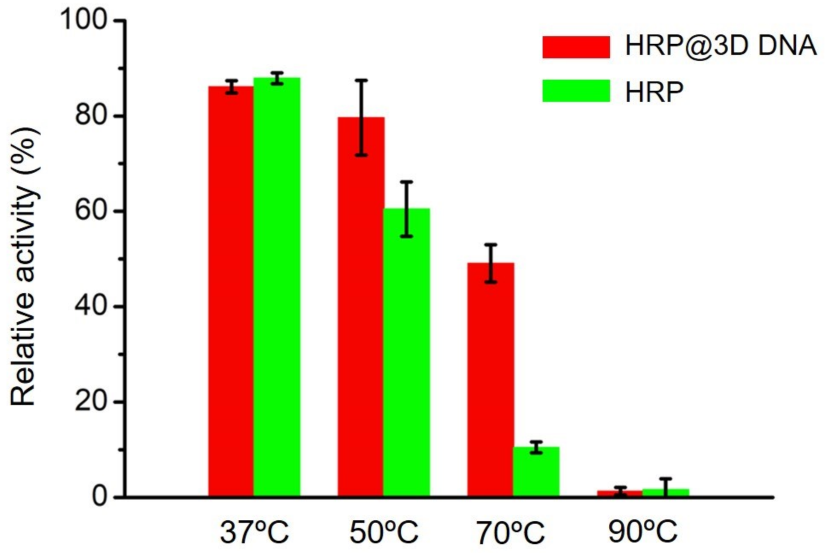

Fig. S22. Loss of HRP activity as a function of temperature. Both HRP@3D DNA and native HRP were assessed.

Experimental details: 5 nM HRP and 2 μL of HRP@3D DNA in 50 μL of ddH_2O were first heated at different temperatures (37°C, 50°C, 70°C, and 90°C) for 5 min. The above solution was mixed with 50 μL of HOAc-NaOAc buffer (0.1 M, pH 4.5) and 5 μL of 20 mM TMB in 96-well plates. 5 μL of 100 mM H_2O_2 was then added before recording the UV-vis absorption at 652 nm. Quantification of the activity was achieved by comparing the initial velocity values of the test samples.

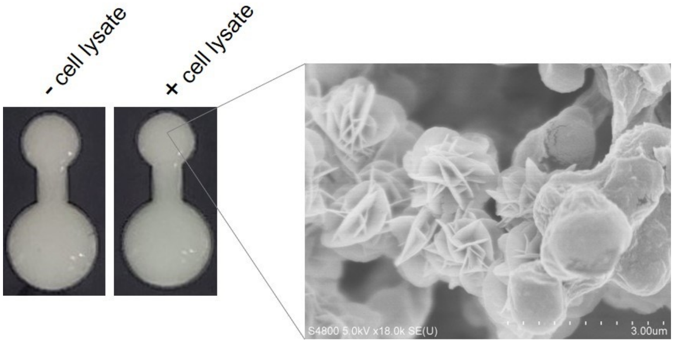

Fig. S23. Treatment of HRP@3D DNA-coated **Z1** with lysate prepared from MCF-7 cells, and SEM image of **Z1** after treatment.

Experimental details: The protocol is similar to the one described in **A3.13** except for: 1) 15 µL of freshly prepared cell lysate was applied to the **Z1**. 2) Cell lysate from 10^6 MCF-7 cells was prepared according to our previously reported method.[1]

References

1 (*a*) M. Liu, J. Song, S. Shuang, C. Dong, J. D. Brennan and Y. Li, *ACS Nano,* 2014, **8**, 5564; (*b*) M. Liu, Q. Zhang, B. Kannan, G. Botton, J. Yang, L. Soleymani, J. Brennan and Y. Li, *Angew. Chem. Int. Ed.,* 2018, **57**, 12440.
